# The TRKB Agonist 7,8-dihydroxyflavone Alleviates DNA Damage and Apoptosis in a Neuronal Cell Model of Friedreich’s Ataxia

**DOI:** 10.1007/s12035-026-05856-2

**Published:** 2026-04-22

**Authors:** Jorge Galán-Cruz, Andrés Vicente-Acosta, Frida Loría, Javier Díaz-Nido, Saúl Herranz-Martín

**Affiliations:** 1 Centro de Biología Molecular Severo Ochoa (CSIC-UAM), Nicolás Cabrera 1, 28049 Madrid, Spain; 2 Departamento de Biología Molecular, Universidad Autónoma de Madrid, Francisco Tomás y Valiente, 7, Ciudad Universitaria de Cantoblanco, 28049 Madrid, Spain; 3 Laboratorio de Apoyo a La Investigación, Hospital Universitario Fundación Alcorcón, Budapest 1, 28922 Alcorcón, Madrid, Spain; 4 Instituto de Investigación Sanitaria Puerta de Hierro - Segovia de Arana, Hospital Universitario Puerta de Hierro, Joaquín Rodrigo 1, 28222 Majadahonda, Madrid, Spain; 5 Departamento de Bioquímica y Biología Molecular, Facultad de Medicina - Universidad Complutense de Madrid, 28040 Madrid, Spain

**Keywords:** Friedreich’s ataxia, 7,8-dihydroxyflavone, DNA damage, Apoptosis, Ferroptosis, Neurodegeneration

## Abstract

Friedreich’s Ataxia (FRDA) is an early onset hereditary disorder with a strong neurodegenerative component caused by repeat expansions on the gene encoding for frataxin (FXN) that result in FXN deficiency. This deficit has been linked to a cascade of biochemical alterations, including mitochondrial dysfunction, oxidative stress and neuronal apoptosis, that drives the neurodegenerative process. FRDA is a very incapacitating disease and patients rely on very limited therapeutic alternatives, such as the recently approved drug omaveloxolone, to treat the oxidative stress. Nevertheless, previous studies have suggested the activation of the brain-derived neurotrophic factor (BDNF) may be a promising treatment to regulate FRDA pathophysiology. Herein, we characterize the effects of FXN deficiency in an in vitro model of primary cerebellar granule neurons (CGNs) derived from the FRDA mouse model YG8-800, as well as the therapeutic potential of BDNF partial agonism by the small molecule 7,8-dihydroxyflavone (7,8-DHF). We found evidence of mitochondrial dysfunction concomitant with DNA damage and enhanced cell death due to FXN deficiency in cultured neurons. The treatment with 7,8-DHF was able to reduce the markers of genotoxicity and apoptosis, without restoring the impaired mitochondrial function nor the total cell death, possibly through ferroptosis, revealing a partial neuroprotective effect insufficient to halt the neurodegenerative process in this in vitro model of FRDA.

**Graphical abstract:**

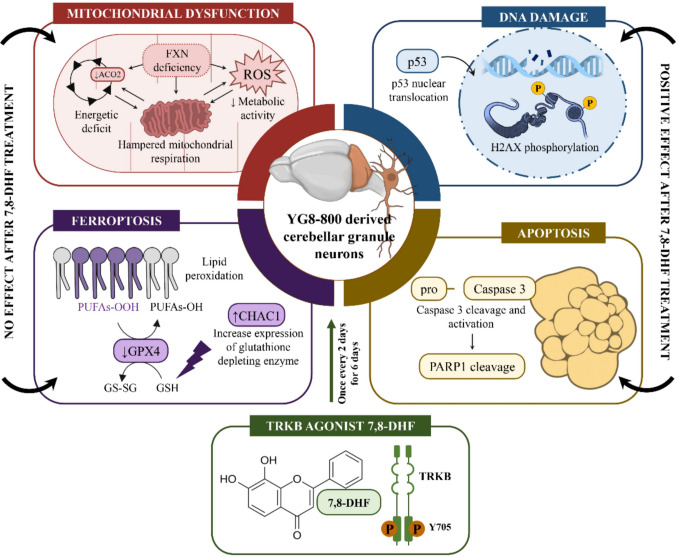

**Supplementary Information:**

The online version contains supplementary material available at 10.1007/s12035-026-05856-2.

## Introduction

Friedreich’s Ataxia (FRDA, OMIM#229300) is an early onset autosomal recessive multisystemic disorder [1] characterized by degeneration of dorsal root ganglia and cerebellar atrophy, hypertrophic cardiomyopathy and musculoskeletal abnormalities [2, 3]. In most cases, FRDA is caused by homozygous repeat expansions of the trinucleotide guanine-adenine-adenine (GAA) in the first intron of the gene encoding for frataxin (FXN) [4]. These mutations can range from 50 to hundreds or thousands of repeats [2] leading to aberrant DNA structures and epigenetic silencing [5], which reduces FXN gene expression to about 25–30% of healthy individuals [3]. FXN is a predominantly mitochondrial protein involved in the regulation of biosynthesis of iron-sulphur (Fe-S) clusters [6, 7], essential inorganic cofactors implicated in oxidative phosphorylation, enzyme catalysis and DNA maintenance, among other functions [8]. Accordingly, cumulative evidence links FXN deficiency to mitochondrial dysfunction, oxidative stress [9, 10], and increased DNA damage [11–13], leading to neuroinflammation and neuronal death [11, 14, 15]. These complex biochemical alterations drive the neurodegenerative process [16].

Until very recently, therapeutic options for FRDA were limited to palliative care [17], but since the beginning of 2023, there is an available treatment for FRDA based on the activation of the nuclear erythroid factor 2 (NRF2) by omaveloxolone [18]. This drug has shown modest positive outcomes in preclinical [19] and clinical studies [20]; however, the severity of FRDA compels to keep developing new treatments. To target different stages of the pathological cascade, several approaches have been proposed, including epigenetic modulators, gene therapy and antioxidants, among many others [17, 19]. Neurotrophins represent a promising alternative, as their therapeutic potential has been widely validated in various neurodegenerative models [21]. In particular, the effects of the brain-derived neurotrophic factor (BDNF) are remarkable, as it is known to regulate neurite outgrowth, synaptic and mitochondrial function and neuron survival [22]. BDNF is enriched in the cerebellum, where it plays a crucial role in the formation, maturation and survival of the granular layer [23]. Furthermore, impaired BDNF expression has been linked to FRDA [24], as well as to other neuropsychiatric disorders, including Alzheimer’s, Huntington’s or Parkinson’s diseases [21].

The development of mouse models that accurately recapitulate the pathophysiology of FRDA is crucial to find new therapies [16]. Some of these models rely on the use of the yeast artificial chromosome (YAC) technology. These models, commonly referred to as YG8, are knockouts for the murine *Fxn* and use the YAC technology to insert the complete human *FXN* gene with a high number of GAA repeats [25]. Various YG8 strains have been developed with increasing number of repeats [17, 25–29], with the most recent model, Fxn^null^::YG8s(GAA)_>800_ (henceforth referred to as YG8-800), harbouring the highest number of triplets, FXN levels closer to those found in patients and the most severe FRDA phenotype reported to date in a mouse model [27–29]. Characterization of the YG8-800 mice revealed a progressive ataxic phenotype resembling that of the human disease, along with reduced aconitase activity, neuroinflammation and behavioural deficits [27–29]. More interestingly, our laboratory has reported a biphasic dynamic of the activation of the BDNF receptor tropomyosin receptor kinase B (TRKB) and BDNF levels [29]. We have observed an upregulation of TRKB activation and BDNF levels in early stages of the disease, when the neurodegeneration has not yet started, followed by a downregulation of both in later stages, concomitant to the neurodegenerative process of the granular and molecular layers of the cerebellum [29].

In light of these findings, we wonder whether BDNF agonism may be a suitable therapy in the YG8-800 FRDA mouse model. Previous studies have demonstrated that BDNF gene therapy is capable of preventing neurodegeneration triggered by FXN deficiency in both in vitro and in vivo FRDA models [30]. Nonetheless, delivering the mature form of BDNF to the central nervous system (CNS) is challenging due to its short half-life and poor bioavailability [31] and normally requires the use of viral vectors [30]. Moreover, chronic BDNF overload in the CNS can lead to adverse effects, including learning deficits or increased neuronal excitability [32]; therefore, BDNF exposure must be tightly regulated. Consequently, the use of a small molecule that mimics BDNF function, permeable to the blood–brain barrier and whose concentration can be more precisely tuned, seems a suitable approach to target neurodegenerative diseases [31]. In this regard, the small molecule 7,8-dihydroxyflavone (7,8-DHF) appears as a potential substitute to BDNF. 7,8-DHF is a partial TRKB agonist that displays better pharmacokinetics than BDNF [33]. Since its discovery, it has been tested in many preclinical models of Alzheimer’s disease, Parkinson’s disease, Huntington’s disease, stroke or spinocerebellar ataxias [33–37], where it improved neuronal and mitochondrial activity [36] and cell survival [31, 33], processes that are also affected in FRDA [16, 38]. Consequently, 7,8-DHF seems to be a promising therapeutic alternative for this disease.

In the present work, we characterize an in vitro model of FRDA based on cerebellar granule neurons (CGNs). CGNs have already been used as an in vitro model of FRDA, using a previous version of the YG8 model, the YG8R mouse [39], and also the YG8-800 model to run a drug screening test [40]. We have previously demonstrated that, in the cerebellum of the YG8-800 mice, CGNs undergo a massive degeneration [29], so cultured CGNs derived from YG8-800 mice may be a highly relevant neuronal model to test pharmacological approaches to curb neurodegeneration in FRDA. Herein, we report neuronal mitochondrial impairment, alongside increased DNA damage and cell death by apoptosis and ferroptosis in cultured CGNs. Additionally, we demonstrate that 7,8-DHF treatment partially alleviates FRDA pathophysiological hallmarks in this neuronal model. In this sense, 7,8-DHF decreases markers of DNA damage and apoptosis, but fails to restore the mitochondrial dysfunction nor the markers of ferroptosis to physiological levels.

## Methods

### Animals

Two strains have been used in this study, the Y47R mice used as controls and also generated by YAC technology but with 9 GAA triplets and normal FXN levels, and the FXN-deficient YG8-800 mice. Both were obtained from Jackson Laboratory (strains #031007 and #030395, respectively) and kept in the animal facility of Centro de Biología Molecular Severo Ochoa at 20–24 °C, 55% relative humidity, <350 lx, <65 db, and food and water ad libitum. They were maintained in cages with sensorial enrichments and fed with a commercial diet.

### Genomic DNA Extraction and Genotyping

Only hemizygous YG8-800 for the FXN gene were used, as they have lower FXN levels and seem to recapitulate better the pathophysiology of the disease. The counterpart hemizygous Y47R was also used as a control. Therefore, newborn mice were genotyped to select hemizygous individuals. Genomic DNA was extracted from tissue samples using the NZY Tissue gDNA isolation kit (NZXTech, Cat. No. MB13503) following manufacturer instructions. Genomic DNA concentrations and purity were determined with the NanoDrop One/OneC spectrophotometer (ThermoFisher, Cat. No. ND-ONE-W). Genotyping was performed by quantitative PCR (see below) and specific *FXN* primers were used to distinguish between hemizygous and homozygous mice: CCCCTGATTTGCTGTATGCT and CTCAAGGTCTCCGCACTTG, forward and reverse primers, respectively.

### Cell Culture

Primary CGNs were obtained from Y47R and YG8-800 mice at postnatal days 5–6. Cerebellum dissection was performed in complete HBSS (Hank’s Balanced Salt Solution, Gibco, Cat. No. 24020-117). They were then washed three times with Ca^2+^ and Mg^2+^ free HBSS (Gibco, Cat. No. 14170-112) followed by digestion with trypsin (0.25%, Gibco, Cat. No. 11580626) and DNase I (1 mg/mL, Roche Diagnostics, Cat. No. 11284932001) for 15 min at 37 °C, and mechanically dissociated with flame-stretched glass Pasteur pipettes. Cells were seeded in plates coated with 100 μg/mL poly-L-lysine (Sigma, Cat. No. P7886) or in coverslips coated with 350 μg/mL poly-L-lysine at a density of 200,000–300,000 cells/cm^2^. CGNs were cultured in Neurobasal media (Gibco, Cat. No. 21103-049) supplemented with 2% B27 (Gibco, Cat. No. 17504-044), 480 μM glutamine, 20 mM KCl and penicillin/streptomycin (100 IU/mL, 100 μg/mL). Cultures were maintained at 37 °C, 5% CO_2_ and 95% humidity.

### Treatment

**Fig. 1:**
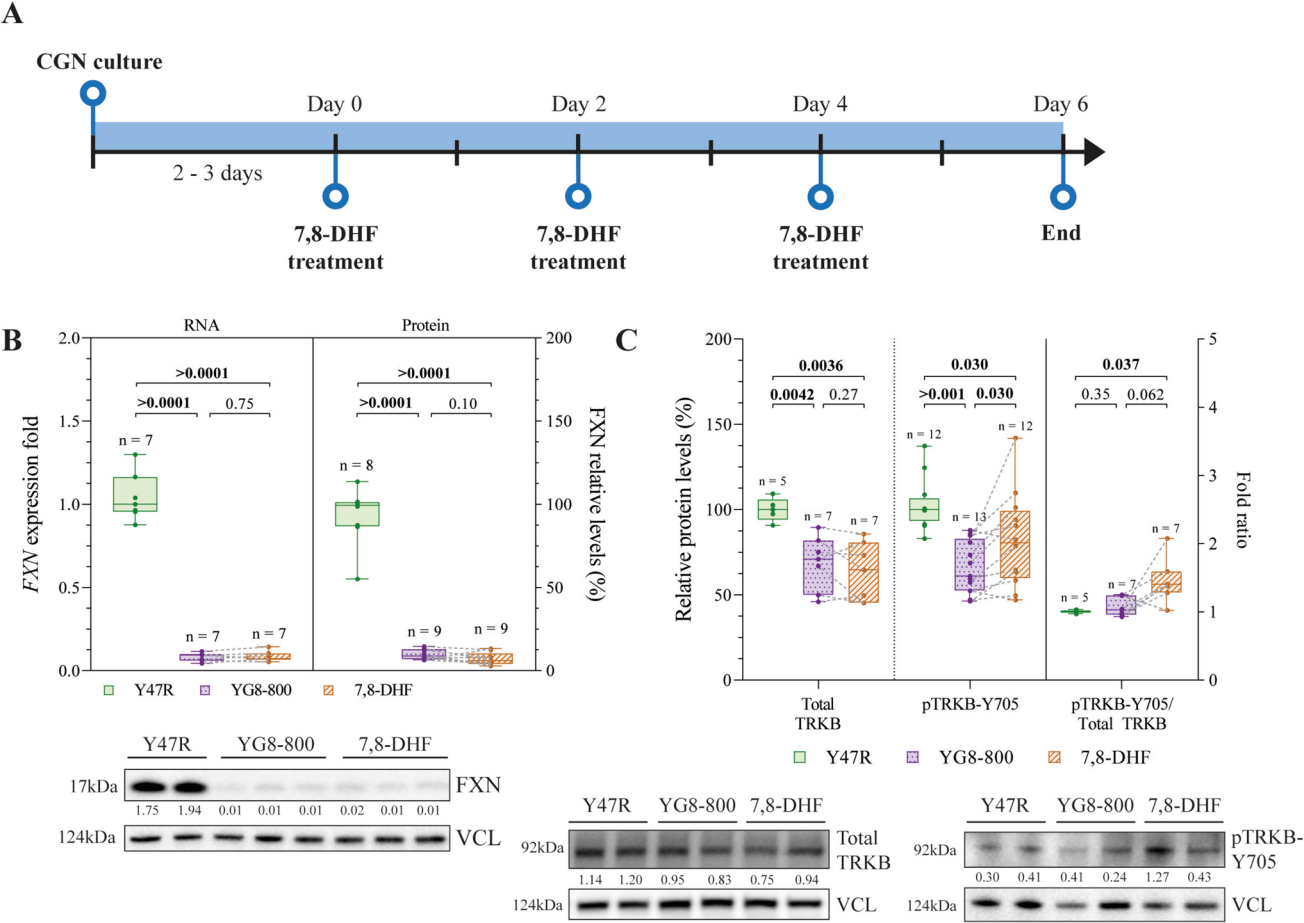
Effect of 7,8-dihydroxyflavone treatment on FXN expression and TRKB activation in cerebellar granule neurons.** A **Experimental design representing the treatment of primary cerebellar granule neurons (CGNs) with the TRKB partial agonist 7,8-dihydroxyflavone (7,8-DHF). **B **Relative gene expression quantification by reverse transcription quantitative PCR of human *FXN* gene (left) and relative levels of FXN protein quantified by Western Blot (right) from Y47R control, YG8-800, and YG8-800 treated with 7,8-DHF primary CGNs. A representative immunoblot is shown below. **C **Relative protein levels of the TRKB receptor (left) and its active phosphorylated form, pTRKB-Y705 (centre), and their ratio (right), quantified by Western Blot from Y47R, YG8-800 and 7,8-DHF treated YG8-800 primary CGNs. Representative immunoblots are shown below. Dots represent CGNs from a single mouse and the dashed line connects matched untreated and 7–8-DHF-treated YG8-800 samples. Data were analysed using a paired Student’s *T*-Test between untreated and 7,8-DHF-treated YG8-800 CGNs (within-mouse comparison) and an unpaired Student’s *T*-Test between Y47R and untreated and 7,8-DHF-treated YG8-800 CGNs. *N* and *p* values are shown in each graph. VCL (vinculin) was used as a loading control and the ratio protein of interest/VCL indicated (**B**, **C**)

The treatments with 7,8-DHF (Sigma-Aldrich, Cat. No. D5446) were performed 2–3 days after seeding; 1 μM of the compound was added once every 2 days for 6 days and cells were collected 48 h since the last 7,8-DHF treatment (Fig. 1A).

### RNA Extraction and Reverse Transcription PCR

Cells were washed once with PBS, frozen in dry ice and stored at −80 °C until processing. Total RNA extraction was performed using the NZY Total RNA Isolation kit (NZYTech, Cat. No. MB13402) following the manufacturer’s instructions, and RNA purity and concentration were determined with the NanoDrop One/OneC spectrophotometer (ThermoFisher, Cat. No. ND-ONE-W). Reverse transcription polymerase chain reaction was performed from 0.5 μg of total RNA (final concentration of 10 ng/μL) using the NZY First-Strand cDNA Synthesis Kit (NZYTech, Cat. No. MB12502) following the manufacturer’s instructions.

### Quantitative PCR

Quantitative PCR (qPCR) was performed from 12 to 20 ng of encoding DNA or 10 ng of genomic DNA using Luminaris Color HiGreen High ROX qPCR Master Mix kit (ThermoFisher, Cat. No. K0363) and CFX Opus 384 Real-Time PCR System (Biorad, Cat. No. 12011452) with the following conditions: 10 min at 95 °C + 40 cycles of 15 s at 95 °C and 1 min at 60 °C + 5 s at 65 °C and increasing 0.5 °C until 95 °C is reached + 5 s at 95 °C. Cycle threshold (Ct) for each gene was normalised with the Ct value of the reference gene Rn18s (ribosomal RNA 18S) and relative expression was calculated by the 2^−ΔΔCt^ [41]. The results are normalised to Y47R samples, and the fold ratio is represented. Primers were used at a final concentration of 500 nM and their efficiency was assumed to be 100%. Primers used are tabulated in the Supplementary Table 1.

### Protein Extraction and Western Blotting

Cells were washed once with PBS, frozen in dry ice and stored at −80 °C. They were then treated with ice-cold RIPA lysis buffer (50 mM Tris–HCl pH of 7.6, 150 mM NaCl, 1% Triton X-100, 0.5% sodium deoxycholate, 0.1% sodic dodecyl sulphate (SDS), 4 mM protease inhibitors (Roche Diagnostics, Cat. No. 11697498001) and 1 μM okadaic acid (phosphatase inhibitor, Sigma-Aldrich, Cat. No. 459618). Each cell well was mechanically disaggregated with cell scrapers and samples were centrifuged 10 min at 13,000 g at 4 °C. Protein concentration was determined using a BCA kit (ThermoFisher, Cat. No. 23227) and samples were denaturalised for 5 min at 100 °C using a loading buffer (10% SDS, 5% β-mercaptoethanol, 0.5% bromophenol blue, 325 mM Tris).

From 5 to 10 µg of protein was loaded and separated by electrophoresis in 4–12% acrylamide-bisacrylamide gradient gels (Invitrogen, Cat. No. NP0322BOX). Proteins were then transferred to nitrocellulose membranes (Invitrogen, Cat. No. IB23002) using the dry transfer method iBlot2 2 (Invitrogen, Cat. No. IB21001), following manufacturer instructions. Membranes were blocked with 5% non-fat dried milk in PBS and 0.1% Tween-20 (Sigma-Aldrich, Cat. No. 822184) and incubated overnight at 4 °C with each primary antibody. Membranes were then incubated for 1 h at room temperature with the specific peroxidase-conjugated secondary antibody. Protein bands were visualized using the chemiluminescence method with the Amersham ECL prime detection system (GE Healthcare Life Science, Cat. No. RPN2232), and protein quantification was performed with ImageJ software. In all cases, values were normalized to the intensity of the loading control vinculin (VCL). The results are normalised to Y47R samples, and the percentage of expression is represented. Primary antibodies are tabulated in the supplementary file S2, Table 2.

### Metabolic Activity Assay

The commercial kit CellTiter96® AQueous One Solution Cell Proliferation Assay, MTS assay (Promega, Cat. No. G3580), was used to determine cell metabolic activity. This is a colorimetric test to quantify the cellular redox state based on the addition of a tetrazolium salt that is reduced to a coloured formazan product by dehydrogenases of metabolically active cells [42]. To perform the assay, the MTS reagent was added at a final concentration of 317 μg/mL to CGNs growth in M96 plates. Once the reagent was added, cells were incubated at 37 °C, with 5% CO_2_ and 95% humidity for 1–2 h and the absorbance at 490 nm was determined using the Dynex Opsys MR microplate reader (Dynex Technologies). To normalize the absorbance by cell number, cells were fixed with 4% paraformaldehyde (PFA) for 15 min, washed with ice-cold PBS and stained with 4′,6-diamidino-2-phenylindole ((DAPI) 1:1000, Merck, Cat. No. 268298) for 10 min. Cell number was quantified with the Agilent BioTek Cytation 5 Cell Imaging Multimode Reader (Agilent Technologies). All final values were normalized to Y47R.

### Mitochondrial Respiration Assay

In order to determine mitochondrial oxygen consumption rate (OCR) and extracellular acidification rate (ECAR) in intact cultured cells [43], the Seahorse XF24 Extracellular Flux Analyzer (Agilent) was used. Seventy-five thousand CGNs were plated in each Seahorse plate well in neuronal media. The assay was performed in Dulbecco’s Modified Eagle Medium (DMEM) supplemented with 5 mM glucose and 1 mM sodium pyruvate, without sodium bicarbonate buffering. Once basal respiration was measured, the ATP synthase inhibitor oligomycin (0.5 μM; Millipore, Cat. No. 495455), the uncoupling agent FCCP (Carbonyl cyanide-p-trifluoromethoxy phenylhydrazone, 0.5 μM; Sigma, Cat. No. C2920) and the mitochondrial complex III inhibitor antimycin A (4 μM; Sigma, Cat. No. A8674) were sequentially added. OCR values were used to determine the maximal respiratory capacity, the basal mitochondrial respiratory capacity, ATP-coupled respiration, proton leak, reserve respiratory capacity and non-mitochondrial respiration [43]. Each mitochondrial parameter was normalized by cell number. This normalization was performed by staining live cells with Hoechst 33258 (1:1000 Merck, Cat. No. TA9H97BAECD2) for 5 min to quantify the cell number using the Agilent BioTek Cytation 5 Cell Imaging Multimode Reader (Agilent Technologies). Results are expressed as a percentage of each Y47R parameter.

### Immunocytochemistry

CGNs were seeded in poly-L-lysine coated glass coverslips placed in multiwell plates. Cells were fixed with 2% PFA for 15 min and 4% PFA for another 15 min. Then, cells were washed three times with ice-cold PBS, blocked with a blocking solution (1% BSA, 0.1% TritonX-100 in PBS 1x) for 20 min at room temperature, and incubated overnight with the primary antibody diluted in the blocking solution. Coverslips were then washed three times with ice-cold PBS, incubated for 1 h at room temperature with the specific secondary antibody conjugated with Alexa-488, Alexa-555 or Alexa-647 (1:1000) diluted in the blocking solution, and washed again three times with ice-cold PBS. After that, cells were stained with DAPI (1:1000, Merck) diluted in PBS and coverslips were mounted on glass slides with Fluoromount G (Southern Biotech Assoc. Inc, Cat. No. 0100-01). To determine any unspecific interactions, a primary antibody control was included. Finally, images were taken with the Laser Scanning Confocal Microscope LSM710 coupled to an inverted AxioObserver microscope (Zeiss). Primary antibodies used are tabulated in the supplementary file S2, Table 2. All quantifications were performed using ImageJ software of at least 4 random fields per sample.

### Cell Viability Assay

Cell viability was determined by measuring calcein-propidium iodide (calcein-PI) uptake. Calcein acetoxymethyl ester is a lipophobic dye that can be taken up by viable cells and then converted by esterases into a hydrophilic form, trapping the dye within the cell; whereas PI is a DNA intercalating agent that can only permeate cells whose membrane has been compromised [44]. This combined staining allows the distinction between healthy and dead cells. CGNs were incubated at 37 °C for 30 min with 2 μM PI (Sigma, Cat. No. P4170) and 1 μM calcein-acetoxymethyl ester (ThermoFisher, Cat. No. C3100MP) and then washed with fresh medium. Four random fields per sample were visualized with an upright Axioskop2 plus microscope (Zeiss) coupled to a colour CMOS camera (DMC6200 Leica) and were analysed in ImageJ. During image taking, cells were maintained at 37 °C and 5% CO_2_. Cell death was expressed as the percentage of PI-positive cells with respect to the total number of cells.

### Lipid Peroxidation Measurement

The commercial probe BODIPY™ 581/591 C11 (ThermoFisher, Cat. No. D3861) was used to measure lipid peroxidation [45]. Cells were cultured in µ-Slide 8 well plates (IBIDI, Cat. No. 80806) and incubated for 30 min with 10 μM BODIPY™ 581/591 C11 at 37 °C. They were washed once, incubated for 15 min with Hoechst (1:1000) at 37 °C and washed again. Finally, lipid peroxidation was determined by measuring the ratio of absorbance at 488 nm (oxidized probe) by 595 nm (reduced probe) of at least 4 fields per condition. Pictures were taken in a Laser Scanning Confocal Microscope LSM800 and analysed in ImageJ. During image taking, cells were maintained at 37 °C and 5% CO_2_.

### Statistical Analyses

The statistical analyses were performed using R v4.1.2 and GraphPad Prism 8.00 software. Unless otherwise specified, data were normalized to the control group Y47R. A paired Student’s *T*-Test was performed between CGNs for each YG8-800 mouse untreated and treated with 7,8-DHF. Then, an unpaired Student’s *T*-Test was performed between Y47R and YG8-800 CGNs, and between Y47R and 7,8-DHF treated YG8-800 CGNs. Finally, the *p*-values for each test were corrected for multiple testing with the Holm-Sidak method with a statistical significance of 0.05. In all figures, the sample size (*n*) of the experiment is specified.

### Ethical Statement

All animal procedures performed were revised and approved by the ethics committee of the Universidad Autónoma de Madrid and by the Community of Madrid (PROEX No 013/021). All efforts were made to minimize the number of the used animals.

## Results

### Treatment with 7,8-DHF Activates TRKB Receptor in FXN-Deficient CGNs

To characterize the effects of the treatment with 7,8-DHF in the pathophysiology mechanisms of FRDA, we established an in vitro model based on CGNs isolated from Y47R and YG8-800 newborns. The latter were treated with 1 μM 7,8-DHF, every other day for a total duration of 6 days (Fig. 1A). The 7,8-DHF dose selection (1 µM) was based on previous literature [37] and unpublished data from our group. First, to evaluate the FXN deficiency of this model, we quantified FXN levels to prove the decrease in FXN transcript and protein levels in YG8-800 neurons compared to controls. This reduction was not recovered by the treatment (Fig. 1B).

As previously mentioned, 7,8-DHF is a TRKB partial agonist [33, 37]. In order to evaluate the activation of TRKB after the treatment, we measured the phosphorylation levels of this receptor in the tyrosine 705 residue (Y705) [46], known to activate this receptor. We observed a reduction in total TRKB and in pTRKB-Y705 levels in untreated YG8-800 CGNs, and the 7,8-DHF treatment recovered the hampered pTRKB-Y705, although not completely comparable to the Y47R condition (Fig. 1C).

To discard potential adverse effects of this treatment, we treated CGNs derived from the control line Y47R with 7,8-DHF. This compound activated the TRKB receptor (Supplementary File S3A and S5) without affecting mitochondrial activity or causing cell death (Supplementary File S3B-C and S5).

Overall, these findings indicate that FXN deficiency in CGNs is concomitant with decreased TRKB levels and diminished TRKB activation. In addition, 7,8-DHF treatment enhances TRKB phosphorylation, but is unable to recover FXN levels.

### YG8-800 CGNs Show Mitochondrial Dysfunction, Which Is Not Recovered by 7,8-DHF Treatment

**Fig. 2:**
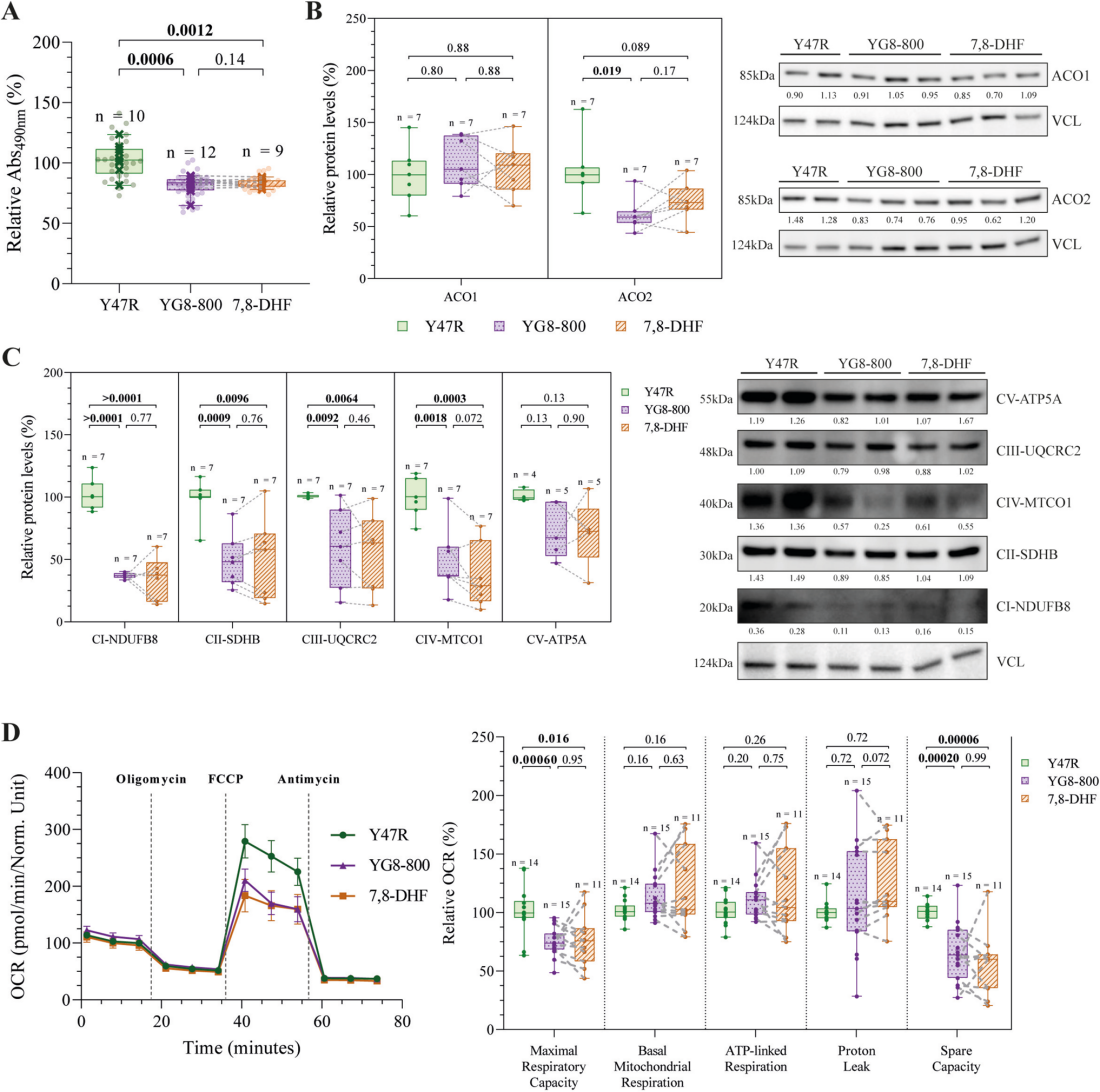
Evaluation of the impact of 7,8-dihydroxyflavone treatment on mitochondrial function.** A **Relative metabolic activity of Y47R control, YG8-800 and 7,8-DHF treated YG8-800 primary cerebellar granule neurons (CGNs). **B **Relative protein levels of the cytosolic isoform of the aconitase ACO1 (left) and its mitochondrial isoform ACO2 (centre) quantified by Western Blot from Y47R, YG8-800 and 7,8-DHF treated YG8-800 primary CGNs. Representative immunoblots are shown on the right. **C **Relative protein levels of some components of the five complexes of the mitochondrial electron transport chain (CI-NDUFB8, CII-SDHB, CIII-UQCRC2, CIV-MTCO1 and CV-ATP5A) quantified by Western Blot from Y47R, YG8-800 and 7,8-DHF treated YG8-800 primary CGNs. Representative immunoblots are shown. **D **Oxygen consumption rate (OCR) quantification by Seahorse between Y47R, YG8-800 and 7,8-DHF treated YG8-800 primary CGNs after the addition of oligomycin, FCCP and antimycin (left). Boxplots in the graph show the quantification of the five mitochondrial parameters derived from the Seahorse assay (right). Dots represent CGNs from a single mouse and the dashed line connects matched untreated and 7–8-DHF-treated YG8-800 samples. Data were analysed using a paired Student’s *T*-Test between untreated and 7,8-DHF-treated YG8-800 CGNs (within-mouse comparison) and an unpaired Student’s *T*-Test between Y47R and untreated and 7,8-DHF-treated YG8-800 CGNs. N and p values are shown in each graph. VCL (vinculin) was used as a loading control and the ratio protein of interest/VCL is indicated (**B**, **C**)

Since FXN has a predominantly mitochondrial function [6], we assessed some metabolic and mitochondrial parameters. First, we quantified the metabolic status of CGNs using the MTS assay (Fig. 2A) to observe a hampered metabolic activity in FXN-deficient neurons compared to controls, which remain unmodified by the treatment. Given the role of FXN in the biosynthesis and maintenance of Fe-S clusters, we measured the levels of several Fe-S-containing proteins, mostly located in the mitochondria. We observed a significant reduction in the mitochondrial isoform of the aconitase (ACO2) in YG8-800 CGNs, an enzyme involved in the tricarboxylic acid cycle. In contrast, the levels for the cytoplasmic isoform of aconitase (ACO1) remained unchanged in YG8-800 CGNs compared to Y47R (Fig. 2B). In addition, a significant reduction in the levels of the mitochondrial transport chain complexes I, II, III, and IV was also found (Fig. 2C). However, no recovery in the levels of any of these mitochondrial proteins was observed after the treatment.

Since a reduction in mitochondrial proteins related to the energy metabolism may correlate with impaired mitochondrial function, mitochondrial respiration was quantified by the Seahorse assay (Fig. 2D). This technique measures the OCR of live cultured cells in response to different modulators of the electron transport chain, allowing the quantification of functional parameters [43]. We detected an overall reduction of the mitochondrial respiration in YG8-800 neurons compared to the control. This was especially prominent in the maximal respiratory capacity, which represents the maximal respiration that a cell can achieve using a mitochondrial uncoupler, emulating a situation with high metabolic demand, and in the spare capacity, which refers to the reserve in mitochondrial respiration that a cell can utilize under a situation of high energy demand [43]. However, we failed to detect any alleviation by the treatment (Fig. 2D).

Furthermore, to corroborate that mitochondrial dysfunction was due to an intrinsic mitochondrial impairment rather than a reduced mitochondrial mass, we assessed mitochondrial content by Western blot analysis of the mitochondrial proteins VDAC1, TOM20 and TIM23, as well as by live-cell imaging using MitoTracker Green (Supplementary File S4 and S5). We did not detect differences in mitochondrial protein levels among CGNs from Y47R, YG8-800 or 7,8-DHF-treated YG8-800 (Supplementary File S4A), nor in mitochondrial mass between CGNs derived from Y47R and YG8-800 mice (Supplementary File S4B).

Overall, these results demonstrate a significant reduction in the levels of several mitochondrial proteins and in the mitochondrial function of FXN-deficient CGNs, without affecting the total mitochondrial mass. However, upon 7,8-DHF treatment, no beneficial effect was observed.

### YG8-800 CGNs Display Increased DNA Damage and Nuclear p53 Levels, Which Are Recovered by 7,8-DHF Treatment

**Fig. 3:**
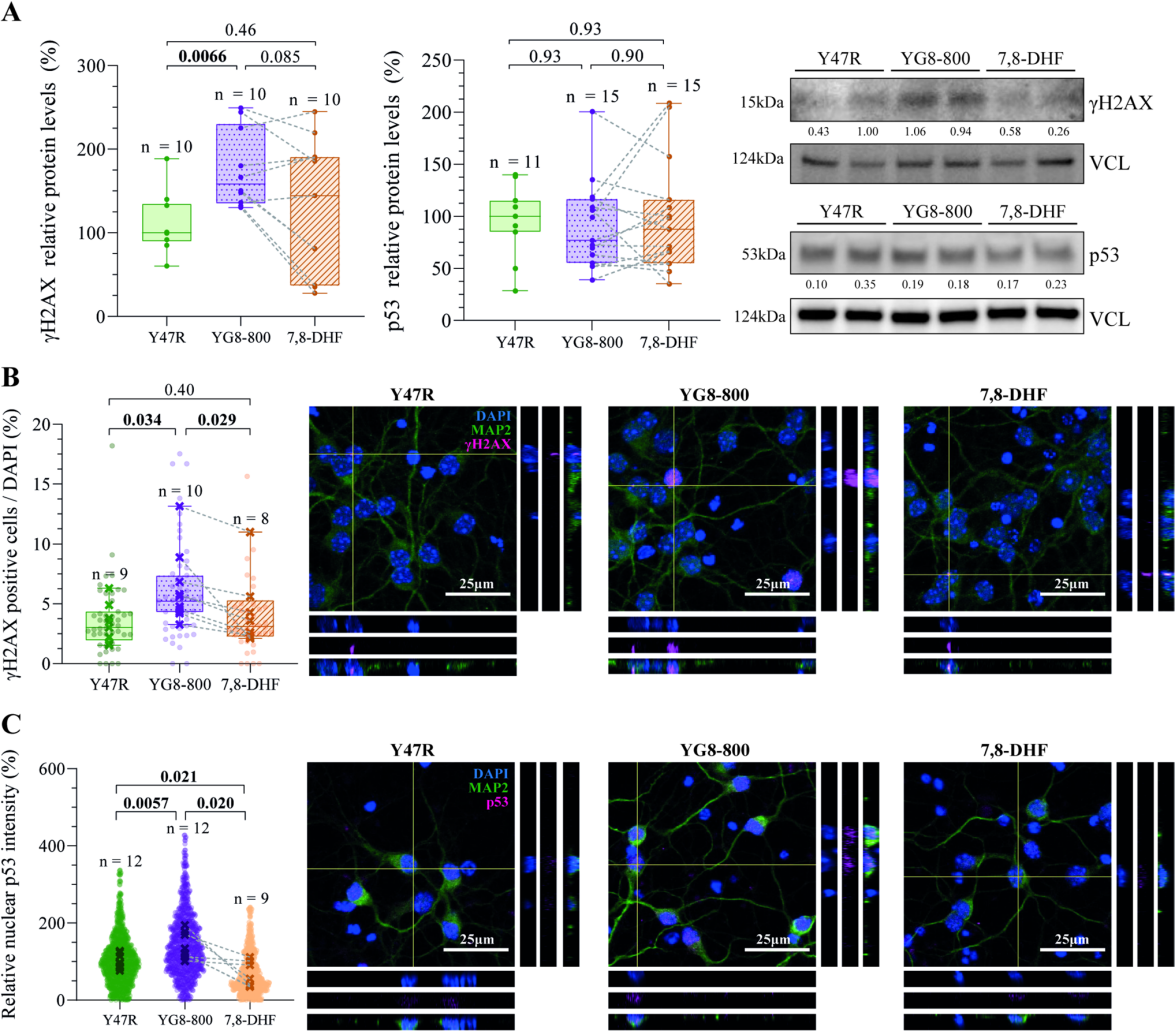
Study of the effect of 7,8-dihydroxyflavone treatment on DNA damage.** A **Relative protein levels of the DNA damage marker γH2AX (left) and p53 (centre) quantified by Western Blot from Y47R control, YG8-800 and 7,8-DHF treated YG8-800 primary cerebellar granule neurons (CGNs). Representative immunoblots are shown (right). **B **Percentage of γH2AX positive cells (magenta) quantified by immunofluorescence from Y47R, YG8-800 and 7,8-DHF treated YG8-800 CGNs cultures (left). Representative images are displayed, including the XZ and YZ cross-sections as insets (right). In the boxplots, dots represent the quantification from a single field, crosses represent the average for each mouse, and the dashed line connects matched untreated and 7–8-DHF-treated YG8-800 samples. **C **Relative levels of nuclear p53 (magenta) fluorescence intensity quantified by immunofluorescence from Y47R, YG8-800 and 7,8-DHF treated YG8-800 CGNs cultures (left). Representative images are displayed, including the XZ and YZ cross-sections as insets (right). Dots represent the quantification of a single cell, crosses represent the average for each mouse, and the dashed line connects matched untreated and 7–8-DHF-treated YG8-800 samples. Data were analysed using a paired Student’s *T*-Test between untreated and 7,8-DHF-treated YG8-800 CGNs (within-mouse comparison) and unpaired Student’s *T*-Test between Y47R and untreated and 7,8-DHF-treated YG8-800 CGNs. *N* and *p* values are shown in each experiment. VCL (vinculin) was used as a loading control and the ratio protein of interest/VCL indicated (A). In blue, the nuclear marker, DAPI, and, in green, the neuronal marker, MAP2, in **B** and **C**

Oxidative stress, mitochondrial failure and loss of DNA repair mechanisms, which are already reported biochemical alterations in FRDA [9–13], have been linked to genotoxic damage [47] and thus, we then evaluated markers of DNA damage in YG8-800 CGNs. Phosphorylation of the H2A histone family member X at Ser139 (γH2AX) is a well-known chromatin modification in response to DNA damage [48], and therefore, we first quantified its protein levels and the number of γH2AX-positive nuclei in our model (Fig. 3A, B). Both parameters were upregulated in untreated YG8-800 compared to Y47R cells. Furthermore, after the treatment, we observed a tendency to reduce γH2AX protein levels (Fig. 3A) and a significantly reduced number of γH2AX-positive nuclei in 7,8-DHF treated cells (Fig. 3B).

To further characterize the effects of 7,8-DHF on the DNA damage response pathway, we quantified p53 levels and translocation to the nucleus as a measure of its activation (Fig. 3A, C). In response to genotoxic damage, p53 is activated and recruited to the nucleus, where it triggers apoptosis in postmitotic cells [49, 50]. Although no difference was observed in total p53 protein levels among conditions (Fig. 3A), a marked increase in the nuclear levels of p53 was identified in YG8-800 untreated CGNs. This nuclear localization was significantly reduced after 7,8-DHF treatment (Fig. 3C).

In summary, these results demonstrate an increase in DNA damage in frataxin-deficient neurons, and a positive effect of our therapy in reducing both DNA damage parameters, the H2AX phosphorylation and p53 nuclear localization, showing a potential beneficial effect of 7,8-DHF treatment in a neuronal frataxin deficiency model.

## YG8-800 CGNs Show Increased Apoptosis and Total Cell Death, Which is Partially Recovered by 7,8-DHF Treatment

**Fig. 4:**
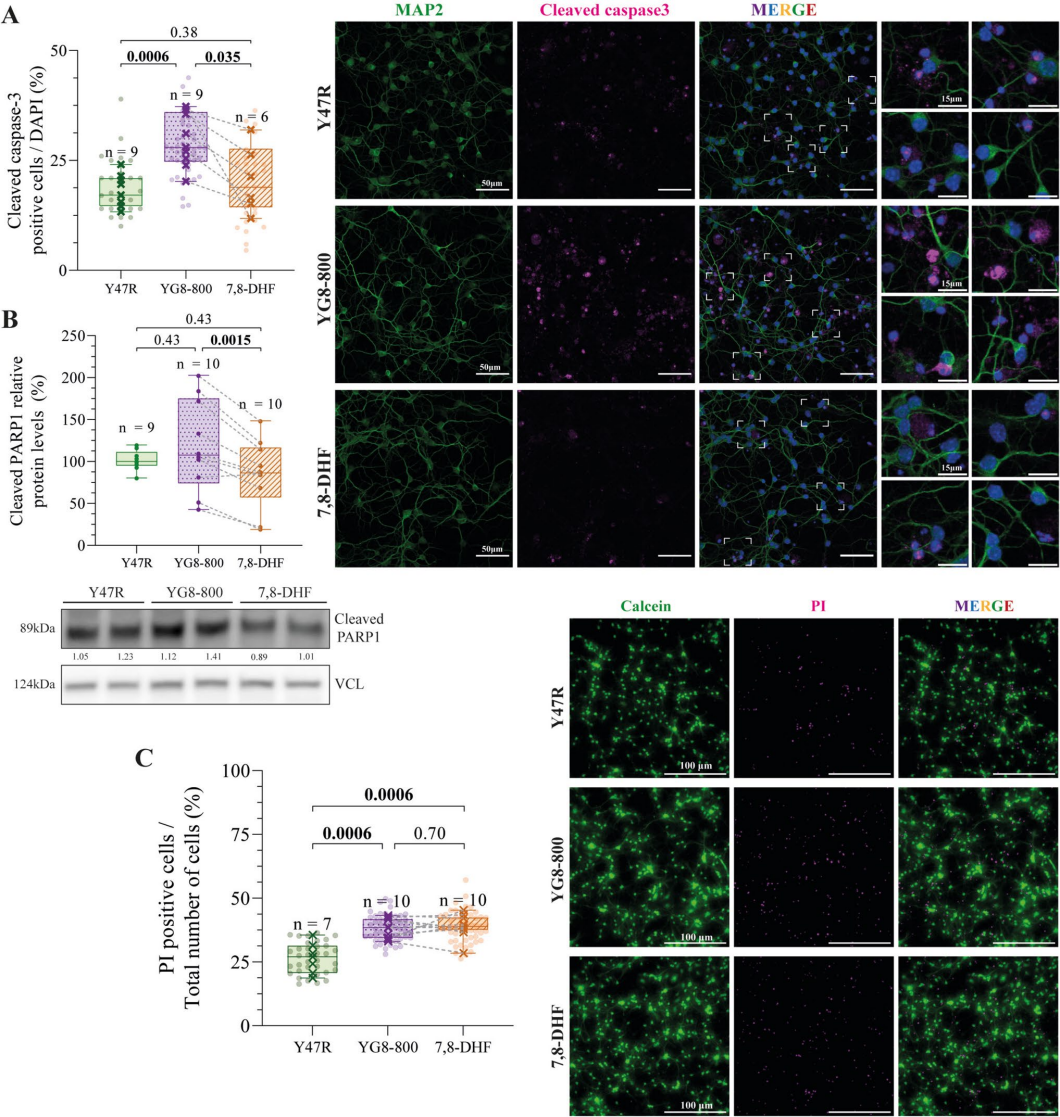
Effect of 7,8-dihydroxyflavone treatment on cell death.** A **Percentage of cleaved caspase-3 positive cells (magenta) quantified by immunofluorescence from Y47R, YG8-800 and 7,8-DHF treated YG8-800 primary cerebellar granule neurons (CGNs) (left). Dots represent the quantification from a single field, crosses represent the average for each mouse, and the dashed line connects matched untreated and 7–8-DHF-treated YG8-800 samples. Representative images are displayed, including magnifications of selected fields as insets (right). **B **Relative protein levels of the cleaved form of PARP1 during apoptosis (at 89 kDa) quantified by Western Blot from Y47R, YG8-800 and 7,8-DHF treated YG8-800 CGN cultures. A representative immunoblot is shown below. **C **Percentage of the cell death marker, propidium iodide (PI, magenta) positive cells after co-staining Y47R, YG8-800 and 7,8-DHF treated YG8-800 CGNs with the live-cell marker, calcein (green) (left). Representative fields are displayed on the right. Dots represent the quantification of a single field, crosses represent the average for each mouse, and the dashed line connects matched untreated and 7–8-DHF-treated YG8-800 samples. Data were analysed using a paired Student’s *T*-Test between untreated and 7,8-DHF-treated YG8-800 CGNs (within-mouse comparison) and unpaired Student’s *T*-Test between Y47R and untreated and 7,8-DHF-treated YG8-800 CGNs. *N* and *p* values are shown in each graph. VCL (vinculin) was used as a loading control and the ratio cleaved PARP1/VCL indicated (**B**). In blue, the nuclear marker, DAPI, and in green, the neuronal marker, MAP2, in **A**

As the activation of p53 can lead to apoptotic death [49, 50], we measured apoptosis and total cell death in FXN-deficient neurons and controls (Fig. 4A–C). Apoptosis was quantified by counting the number of cleaved caspase-3 positive cells and measuring protein levels of the cleaved form of PARP1, a caspase-3 target and a known hallmark of apoptosis (Fig. 4A, B) [51]. We identified an increased number of cleaved caspase-3 positive cells in FXN-deficient neurons, but we did not find a significant difference in the levels of cleaved PARP1 between YG8-800 and Y47R cells. However, 7,8-DHF treatment significantly reduced the number of cleaved caspase-3 positive cells to values comparable to Y47R neurons, as well as the levels of PARP-1 compared to untreated YG8-800 cells, evidencing a positive effect of 7,8-DHF on the apoptotic cascade (Fig. 4A, B).

When the total cell death was evaluated through the calcein-PI method, we observed a significant increase in the number of PI positive cells in YG8-800 CGNs (Fig. 4C). However, this increase in cell death was not rescued by the treatment (Fig. 4C). These results suggest that while 7,8-DHF may block the apoptotic cascade, there is an additional mechanism that drives cell death in FXN-deficient CGNs.

### 7,8-DHF Does Not Improve the Lipid Peroxidation and Altered Ferroptosis Markers

**Fig. 5:**
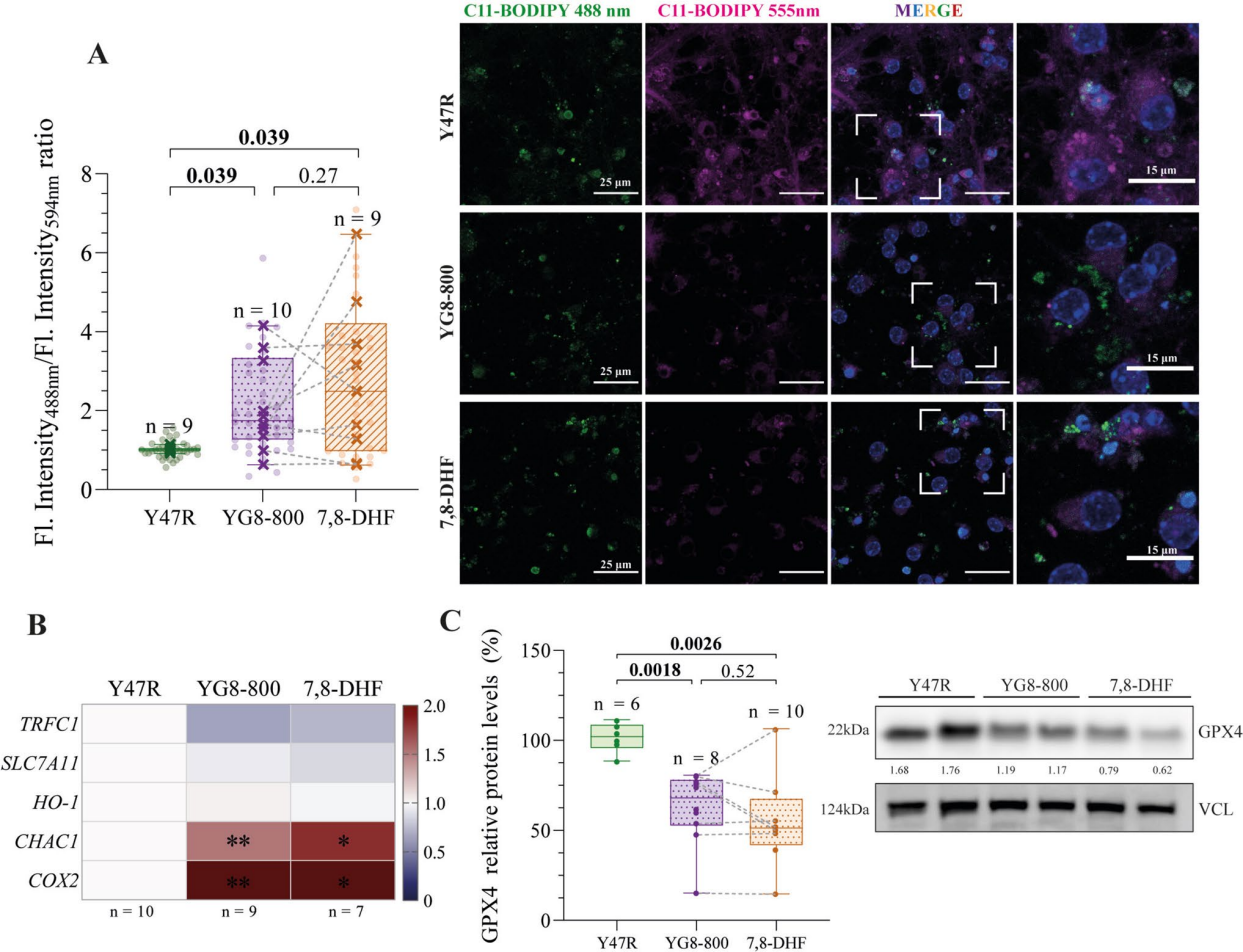
Assessment of 7,8-dihydroxyflavone treatment on lipid peroxidation and ferroptosis.** A **Ratio of the fluorescence intensity of the C11-BODIPY probe sensitive to lipid peroxidation at 488 nm (oxidised probe in green) by the fluorescence intensity at 594 nm (reduced probe in magenta) by live-cell imaging from Y47R control, YG8-800 and 7,8-DHF treated YG8-800 primary cerebellar granule neurons (CGNs) (left). Dots represent the quantification from a single field, crosses represent the average for each mouse, and the dashed line connects matched untreated and 7–8-DHF-treated YG8-800 samples. Representative images are displayed, including magnifications of selected fields as insets (right). **B **Heatmap of the relative gene expression of several genes involved in ferroptosis quantified by reverse transcription quantitative PCR of Y47R, YG8-800 and 7,8-DHF treated YG8-800 CGNs. A downregulation from the control, which is set to 1.0, is represented in blue and an upregulation is represented in red. Median values of each condition are represented in each cell. **C **Relative protein levels of GPX4 quantified by Western Blot from Y47R, YG8-800 and 7,8-DHF treated YG8-800 CGN cultures (left). A representative immunoblot is shown below, using VCL as a loading control (right). Dots represent CGNs from a single mouse and the dash line connects matched untreated and 7–8-DHF-treated YG8-800 samples. In **A** and **C**, data were analysed using a paired Student’s *T*-Test between untreated and 7,8-DHF-treated YG8-800 CGNs (within-mouse comparison) and unpaired Student’s *T*-Test between Y47R and untreated and 7,8-DHF-treated YG8-800 CGNs. In **B**, data were evaluated by mixed-effects model ANOVA test, setting the significance at *p < 0.05 and **p < 0.01. *N* and *p* values are shown in each graph. VCL (vinculin) was used as a loading control and the ratio GPX/VCL indicated (**C**). In blue, the nuclear marker, Hoechst, in **A**

Several models of FRDA have been linked to increased activation or increased sensitivity to ferroptosis [19, 52], an iron-dependent cell death [53, 54]. Therefore, we wondered whether the discrepancy between the reduced apoptosis and sustained cell death in the 7,8-DHF-treated YG8-800 was due to ferroptosis. Consequently, we measured some markers associated with this type of cell death in our model (Fig. 5A–C). We first analysed the intensity of the C11-BODIPY probe (Fig. 5A), a fluorescent dye used as an indicator of lipid peroxidation, one of the hallmarks of ferroptosis [45]. An increased ratio of 488 nm (oxidized probe by lipid peroxides) to 595 nm fluorescence (reduced probe) was observed in YG8-800 CGNs compared to Y47R cells, indicating an accumulation of lipid peroxides. However, we did not detect a consistent alleviation by the treatment (Fig. 5A). Indeed, we observed inter-individual variability when comparing untreated and 7,8-DHF-treated matched CGNs samples, while the treatment reduced lipid peroxide levels in some animals, levels remained stable or increased in others (as indicated by the dashed lines in Fig. 5A).

To better understand the contribution of this type of cell death in our model, the expression of some markers linked to ferroptosis was measured (Fig. 5B). We observed an upregulation of some of these markers, including ChaC glutathione specific gamma-glutamylcyclotransferase 1 (*Chac1*), a glutathione depleting enzyme [54]; and cyclooxygenase-2 (*Cox2*), a ROS producing enzyme upregulated in response to ferroptosis stimuli [54]. However, the treatment with 7,8-DHF did not affect the expression of any of these markers.

Finally, we assessed the protein levels of the glutathione peroxidase 4 (GPX4), an antioxidant enzyme involved in the reduction of lipid peroxides and whose downregulation has been associated with ferroptosis [53, 54] and with models of FRDA [55]. In our case, we observed reduced GPX4 protein levels in YG8-800 CGNs compared to controls, which were not restored by 7,8-DHF treatment (Fig. 5C). Furthermore, as previously observed, there is a marked inter-individual variability among untreated and 7,8-DHF-treated matched CGNs samples in relation to GPX4 protein expression following the treatment (dashed lines in Fig. 5C).

Taken together, these results demonstrate that YG8-800 CGNs display increased lipid peroxidation and altered levels of markers associated with ferroptosis. The treatment with 7,8-DHF did not restore either of the two parameters, suggesting that it is incapable of halting ferroptosis in this FXN-deficiency model.

## Discussion

FRDA is a neurodegenerative disease [1] with very limited therapeutic options available, with the exception of omaveloxolone, and patients usually rely on palliative care [17, 18, 20]. In this regard, BDNF agonism emerges as an interesting approach for FRDA treatment. BDNF is known to enhance mitochondrial function and prevent apoptosis [21, 22]. Furthermore, dysregulation of the BDNF pathway has been linked to many neurodegenerative diseases, including FRDA [21, 24]. Previous studies have shown a potential therapeutic effect of BDNF gene therapy in other FRDA models [30]. However, BDNF has poor pharmacokinetic properties [33], and therefore, the small molecule 7,8-DHF, a partial agonist of the BDNF receptor TRKB, has emerged as a therapeutic agent. Since 7,8-DHF has been widely evaluated in several neurodegenerative preclinical models with promising results [31, 33–36], we decided to test its efficacy in CGNs cultured from YG8-800 mice, a model for FRDA. This neuronal population was selected because CGNs are the most severely affected neuronal cell type in vivo within the YG8-800 mouse cerebellum [29].

7,8-DHF promotes TRKB activation by phosphorylating its Y705 [46]. Previously, we have observed a hampered activation of TRKB in FXN-deficient neurons, consistent with the defective TRKB phosphorylation in the cerebellum of YG8-800 mice at advanced stages of the disease [29]. In the present study, we have shown that 7,8-DHF treatment partially restored TRKB phosphorylation in our FXN-deficient CGNs, corroborating its efficacy as a TRKB partial agonist.

As mentioned above, CGNs derived from YG8-800 mice display FXN transcript and protein levels of around 5–10% of healthy controls, similar values to those observed in human patients [4]. Since the deficiency in these mice is caused by an epigenetic silencing of the human FXN gene, the etiology is comparable to that of the human disease [5]. FXN is a predominantly mitochondrial protein [6, 7], whose deficiency impacts the levels of Fe-S cluster-containing enzymes, resulting in hampered mitochondrial respiration [15, 38] and a fragmented mitochondrial phenotype [56]. While fission events remain to be elucidated in this model, our results corroborate a marked metabolic deficit along with reduced mitochondrial respiration, with significant impairments in the maximal respiratory and spare capacities. Decreased levels of Fe-S cluster-containing enzymes, such as ACO2 and several complexes of the electron transport chain were also described. These findings are consistent with previous studies in both patient-derived cells and FRDA models [4, 9, 15, 27–29, 38]. Furthermore, a defective electron transport chain leads to oxidative stress [7], explaining the reduced metabolic activity observed in our in vitro model. However, after the 7,8-DHF treatment, we did not find any positive effect on mitochondrial or metabolic parameters in FXN-deficient CGNs. This may be because the treatment did not restore FXN levels and thus, the deficiency of Fe-S clusters remains and the function of proteins containing these cofactors is still impaired.

Diminished mitochondrial metabolism, in a context of genetic instability, is characteristic of FRDA patients [2, 3] and may contribute to DNA damage. DNA damage has been reported in other neurodegenerative diseases, where it mediates neuronal death [57]. In FRDA, downregulation of several DNA repair pathways has been described alongside an accumulation of DNA lesions [11–13]. In our study, we observed increased genotoxic damage reflected as the number of γH2AX foci in YG8-800-derived CGNs. H2AX is a histone variant responsible for recruiting the DNA repair machinery following a double strand break [48]. In this FXN-deficiency model, DNA damage may not be properly repaired, leading to cell death and neurodegeneration. In other cell types, such as myoblasts, 7,8-DHF has been shown to act as an antioxidant agent, attenuating acute DNA damage through a mechanism involving *Ho-1* upregulation [58]. However, in our YG8-800-derived CGNs, 7,8-DHF reduced γH2AX foci without increasing *Ho-1* transcript levels. Further studies are needed to determine whether this difference is due to cell-type specific differences or to the prolonged ROS exposure in FXN-deficient CGNs. Indeed, although direct measurement of ROS species was not performed in this study, given that oxidative stress is a primary driver of DNA damage [59], future studies should clarify the link between ROS generation and genotoxic damage in the YG8-800 model. Furthermore, the oxidative stress profile observed in the YG8-800 CGNs compared to controls suggests that oxidative DNA damage could likely drive the observed DNA double-strand breaks detected in this study. Further analyses addressing the levels of markers such as 8-hydroxy-2′deoxygaunosine (8-OHdG) will be essential to determine the role of oxidative modifications in the genomic instability of this model.

Furthermore, a genotoxic signalling cascade could lead to an activation of p53 and apoptosis, and thus, we then evaluated the dynamics of this protein. p53 responds to oxidative stress and DNA damage by translocating to the nucleus, where it regulates the expression of genes involved in apoptosis in postmitotic cells [49]. FRDA and FXN deficiency have been associated with increased p53 transcript and protein levels [12, 14, 50], suggesting an upregulation of the DNA damage response. In our study, we observed enhanced translocation of p53 to the nuclear area in YG8-800-derived CGNs, without an increase in total protein levels. As many of the studies associating increased levels of p53 with FXN deficiency are performed in models with an acute and severe loss of FXN [12, 14, 50], the difference could lie in the chronic deficiency exhibited by these CGNs. In previous models, the sudden reduction in mitochondrial function could lead to a significant increase in ROS production, which explains the enhanced p53 expression. In the YG8-800 model, the CGNs are adapted to the genetic FXN deficiency and consequently, p53 levels are not significantly increased. Instead, p53 translocates to the nucleus in response to the continuous damage produced by the mitochondrial dysfunction. In this regard, 7,8-DHF treatment was found to effectively reduce nuclear p53 localization. Additionally, p53 levels can also be regulated by pMDM2, a downstream effector of the PI3K/Akt pathway, which is also activated by 7,8-DHF [58]. Therefore, further studies are required to clarify the precise mechanism by which this compound provides neuroprotection in CGNs.

As previously mentioned, p53 is a well-known mediator of apoptosis [49]. Indeed, apoptotic cell death has been widely associated with a plethora of neurodegenerative diseases [60]. In FRDA, extensive literature links FXN deficiency to upregulation of the apoptotic cascade in several in vitro models, [12, 14, 50, 61–63]. Therefore, we characterize some well-known apoptosis markers, such as caspase-3 and PARP1. Caspase-3 is an effector caspase which is cleaved and activated by initiator caspases, whereas PARP1 cleavage into an 85 kDa fragment is specifically generated by caspase-3 activity [51]. In our model, increased caspase-3 was observed by immunocytochemistry in YG8-800 CGNs, but PARP1 cleavage was not significantly enhanced, possibly due to a lack of sensitivity of the Western blot. Nevertheless, caspase-3 cleavage is concomitant with enhanced cell death, supporting an extensive neurodegenerative process. Moreover, several studies have demonstrated the potential anti-apoptotic effect of 7,8-DHF in in vitro models of various neurodegenerative diseases [64]. In agreement with those results, in our study, the levels of apoptosis markers in FXN-deficient CGNs were reduced after the treatment with 7,8-DHF. However, when total cell death was analysed, we found no significant reduction with the treatment, suggesting that while 7,8-DHF can reduce apoptosis, it cannot ultimately reverse the neurodegenerative process.

To understand the gap between the reduced apoptosis and maintained cell death, we looked into other types of cell death, as ferroptosis. This type of cell death is biochemically defined by an accumulation of intracellular iron that results in increased lipid peroxidation [53]. Increased lipid peroxidation, iron dysregulation and oxidative stress have been reported in in vitro and in vivo FRDA models [65]. In accordance with that, we have recently shown that the cerebellum of YG8-800 mice accumulates iron [29]. Although we have not directly evaluated the iron levels in our in vitro model, we have found increased lipid peroxidation in FXN-deficient CGNs. In order to analyse the ferroptopic profile of the FXN-deficient CGNs, we have also observed a reduction in the levels of the enzyme GPX4, a well-known inhibitor of ferroptosis that reverts lipid peroxidation [53]. Moreover, we have detected an upregulation of several markers for ferroptosis, including the *Chac1* and *Cox2* genes [53]. Remarkably, we did not find any positive effect of the 7,8-DHF treatment on reverting any of the altered parameters associated with ferroptosis, suggesting that this drug could potentially prevent apoptosis, but it does not have any effect on the ferroptotic cascade. This would finally lead to the same rate of cell death as the untreated FXN-deficient CGNs, as our study proved.

In summary, these results show that CGNs derived from the YG8-800 mouse model recapitulate many of the biochemical alterations reported in other FRDA models and in patients. In this sense, we have demonstrated that these YG8-800-derived CGNs exhibit FXN deficiency, similar to FRDA patients, as well as reduced TRKB activation. Moreover, we found reduced levels of several mitochondrial proteins involved in the energetic metabolism that leads to mitochondrial and metabolic dysfunction in FXN-deficient CGNs. In addition to this, increased DNA damage and p53 translocation to the nucleus were also reported, along with enhanced cell death driven by apoptosis and ferroptosis, suggesting an interplay of both processes in the pathophysiology of this model of FRDA. Finally, we have also demonstrated the therapeutic potential of the TRKB partial agonist 7,8-DHF on this model. We found that 7,8-DHF was capable of recovering the attenuated TRKB activation and ameliorated both the markers of DNA damage and apoptosis. Therefore, we hypothesize that 7,8-DHF is able to block the apoptotic cascade in this model. However, the FXN deficiency is not reverted by the treatment and thus, the mitochondrial dysfunction persists, driving the neurodegenerative process. Since previous studies have indicated that 7,8-DHF is only a partial and not full agonist of TRKB [37], further studies are required to determine whether or not full BDNF agonism is able to prevent neurodegeneration in CGNs.

## Supplementary Information

Below is the link to the electronic supplementary material.

ESM 1 (DOCX 15.0 KB)

ESM 2 (DOCX 15.6 KB)

ESM 3 Effect of 7,8-dihydroxyflavone treatment on CGNs derived from control Y47R mice. **A**—Relative protein levels of the TRKB receptor, its active phosphorylated form, pTRKB-Y705, and the ratio between both, quantified by Western Blot, from Y47R 7,8-DHF-treated CGNs. Representative immunoblots are shown on the left. **B**—Percentage of the cell death marker, propidium iodide (PI, magenta) positive cells after co-staining Y47R 7,8-DHF-treated CGNs with the live-cell marker, calcein (green). Dots represent the quantification from a single field and crosses represent the average for each mouse. Representative fields are displayed below. C- Relative metabolic activity of Y47R 7,8-DHF-treated CGNs measured by the MTS assay. Dots (**A** and **C**) and crosses (**B**) represent CGNs from a single mouse and the dash line connects matched untreated and 7–8-DHF-treated Y47R samples (**A** and **B**). Data were analysed using a paired Student’s T-Test between untreated and 7,8-DHF-treated Y47R CGNs and p values are shown in each graph. VCL (vinculin) was used as a loading control in A. (PNG 894 KB)

ESM 3 High Resolution Image (TIF 28.3 MB)

ESM 4 Measurement of mitochondrial mass. **A**—Relative protein levels of the mitochondrial proteins VDAC1, TIM23 and TOM20, quantified by Western Blot, from Y47R and YG8-800 untreated or 7,8-DHF-reated CGNs. Dashed lines connect matched untreated and 7–8-DHF-treated YG8-800 samples. Representative immunoblots are shown on the left. **B** – Analysis of the mitochondrial network, using the ImageJ plugin Mitochondrial Network Analysis (MiNA), upon green mitotracker staining. Representative live-cell images are shown on the right. Dots represent the quantification from a single field, and crosses represent the average for each mouse, Data were analysed using a paired Student’s T-Test between untreated and 7,8-DHF-treated YG8-800 CGNs (within-mouse comparison) and unpaired Student’s T-Test between Y47R and untreated and 7,8-DHF-treated YG8-800 CGNs. N number and p values are shown in each graph. VCL (vinculin) was used as a loading control in **A**. In blue, the nuclear marker, Hoestch, in **B**. (PNG 1.15 MB)

ESM 4 High Resolution Image (TIF 21.8 MB)

ESM 5 (DOCX 14.0 KB)

## Data Availability

All the data supporting the current study are available from the corresponding author upon reasonable request.

## Abbreviations

7,8-DHF: 7,8-Dihidroxyflavone
BDNF: Brain-derived neurotrophic factor
CGNs: Cerebellar granule neurons
CNS: Central nervous system
DAPI: 4′,6-Diamidino-2-phenylindole
DMEM: Dulbecco’s Modified Eagle Medium
Fe-S: Iron-sulphur
FRDA: Friedreich’s ataxia
FXN: Frataxin
GAA: Guanine adenine adenine
GPX4: Glutathione peroxidase 4
γH2AX: Phosphorylated histone H2A family member X
OCR: Oxygen consumption rate
PFA: Paraformaldehyde
PI: Propidium iodide
TRKB: Tropomyosin receptor kinase B
VCL: Vinculin
YAC: Yeast artificial chromosome
