# Supplementary material for: The TRKB Agonist 7,8-dihydroxyflavone Alleviates DNA Damage and Apoptosis in a Neuronal Cell Model of Friedreich’s Ataxia
Source: Mol Neurobiol. 2026 Apr 22;63(1):580. doi: 10.1007/s12035-026-05856-2 (PMC13102869; doi:10.1007/s12035-026-05856-2)
Supplement: Supplementary file 1 — (DOCX 15.0 KB) [file 12035_2026_5856_MOESM1_ESM.docx]

**Supplementary file S1: Table 1. List of qPCR primers**

| Gen | Forward primer | Reverse primer | Primers efficiency |
| --- | --- | --- | --- |
| *Chac1* | CTGTGGATTTTCGGGTACGG | CCCCTATGGAAGCTGTCTCC | 93% |
| *Cox2* | TTCAACACACTCTATCACTGGC | AGAAGCGTTTGCGGTACTCAT | 109% |
| *FXN* | TGGAATGTCAAAAAGCAGAGT | CCACTCCCAAAGGAGACATC | 94% |
| *Ho1* | CACTCTGGAGATGACACCTGAG | GTGTTCCTCTGTCAGCATCACC | 107% |
| *Rn18s* | GCAATTATTCCCCATGAACG | GGGACTTAATCAACGCAAGC | 91% |
| *Slc7a11* | TGGGTGGAACTGCTCGTAAT | AGGATGTAGCGTCCAAATGC | 94% |
| *Trfc1* | AATGGTTCGTACAGCAGCGGAAG | TAGCACGGAAGTAGTCTCCACGAG | 98% |
