# Supplementary material for: The TRKB Agonist 7,8-dihydroxyflavone Alleviates DNA Damage and Apoptosis in a Neuronal Cell Model of Friedreich’s Ataxia
Source: Mol Neurobiol. 2026 Apr 22;63(1):580. doi: 10.1007/s12035-026-05856-2 (PMC13102869; doi:10.1007/s12035-026-05856-2)
Supplement: Supplementary file 2 — (DOCX 15.6 KB) [file 12035_2026_5856_MOESM2_ESM.docx]

**Supplementary file S2: Table 2. List of primary antibodies**

| Protein | Source | Reference | Dilution |
| --- | --- | --- | --- |
| ACO1 | Rabbit | Abcam #ab126595 | 1:1000 (WB) |
| ACO2 | Rabbit | Abcam #ab71440 | 1:1000 (WB) |
| Cleaved caspase-3 | Rabbit | Cell signaling #9661S | 1:400 (IF) |
| FXN | Mouse | Abcam #ab110328 | 1:1000 (WB) |
| γH2AX | Rabbit | Cell signaling #9718 | 1:500 (IF) |
| GPX4 | Rabbit | Abcam #ab125066 | 1:1000 (WB) |
| p53 | Mouse | Cell signaling #2524 | 1:1000 (WB); 1:1000 (IF) |
| PARP1 | Rabbit | Abcam #ab191217 | 1:1000 (WB) |
| TRKB | Rabbit | Santa Cruz biotechnology #sc12 | 1:1000 (WB) |
| pTRKB-Y705 | Rabbit | GenScript A01186 | 1:1000 (WB) |
| VCL | Mouse | Sigma-Aldrich #CP74 | 1:2000 (WB) |
