# Supplementary figures and images for: The TRKB Agonist 7,8-dihydroxyflavone Alleviates DNA Damage and Apoptosis in a Neuronal Cell Model of Friedreich’s Ataxia
Source: Mol Neurobiol. 2026 Apr 22;63(1):580. doi: 10.1007/s12035-026-05856-2 (PMC13102869; doi:10.1007/s12035-026-05856-2)

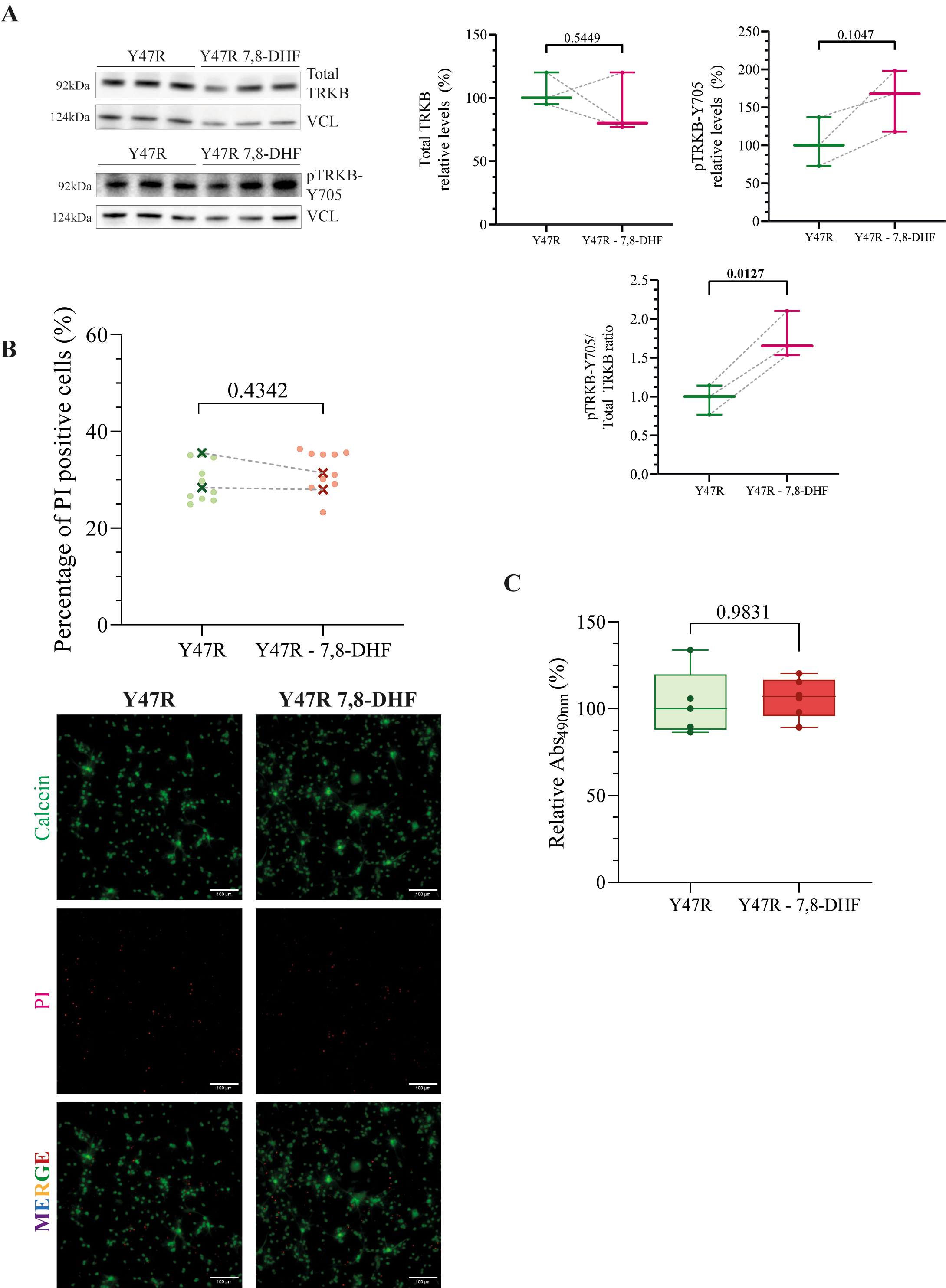

Supplement: Supplementary file 3 — Effect of 7,8-dihydroxyflavone treatment on CGNs derived from control Y47R mice. A—Relative protein levels of the TRKB receptor, its active phosphorylated form, pTRKB-Y705, and the ratio between both, quantified by Western Blot, from Y47R 7,8-DHF-treated CGNs. Representative immunoblots are shown on the left. B—Percentage of the cell death marker, propidium iodide (PI, magenta) positive cells after co-staining Y47R 7,8-DHF-treated CGNs with the live-cell marker, calcein (green). Dots represent the quantification from a single field and crosses represent the average for each mouse. Representative fields are displayed below. C- Relative metabolic activity of Y47R 7,8-DHF-treated CGNs measured by the MTS assay. Dots (A and C) and crosses (B) represent CGNs from a single mouse and the dash line connects matched untreated and 7–8-DHF-treated Y47R samples (A and B). Data were analysed using a paired Student’s T-Test between untreated and 7,8-DHF-treated Y47R CGNs and p values are shown in each graph. VCL (vinculin) was used as a loading control in A. (PNG 894 KB) [file 12035_2026_5856_Fig6_ESM.png]

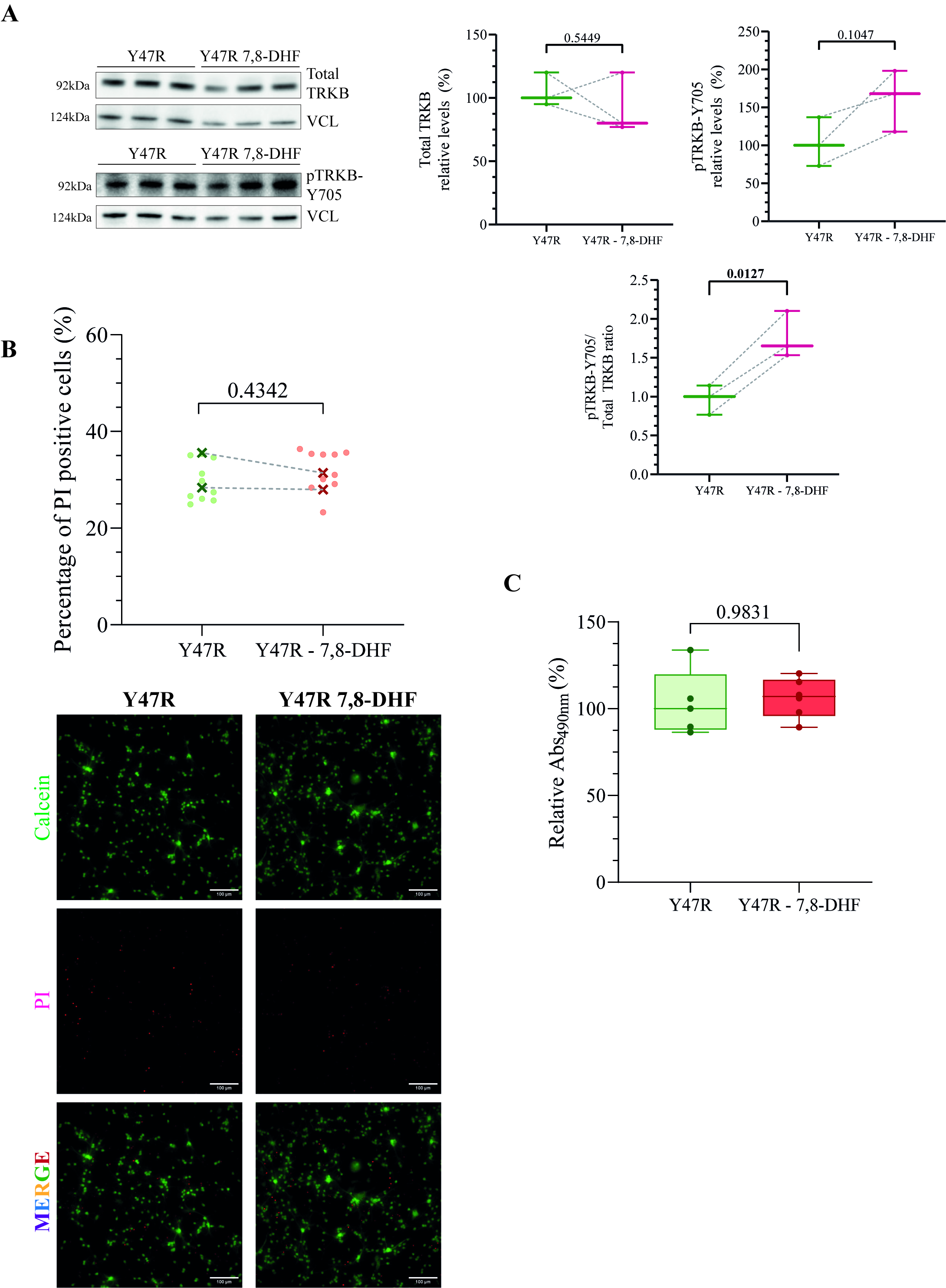

Supplement: Supplementary file 4 — High Resolution Image (TIF 28.3 MB) [file 12035_2026_5856_MOESM3_ESM.tif]

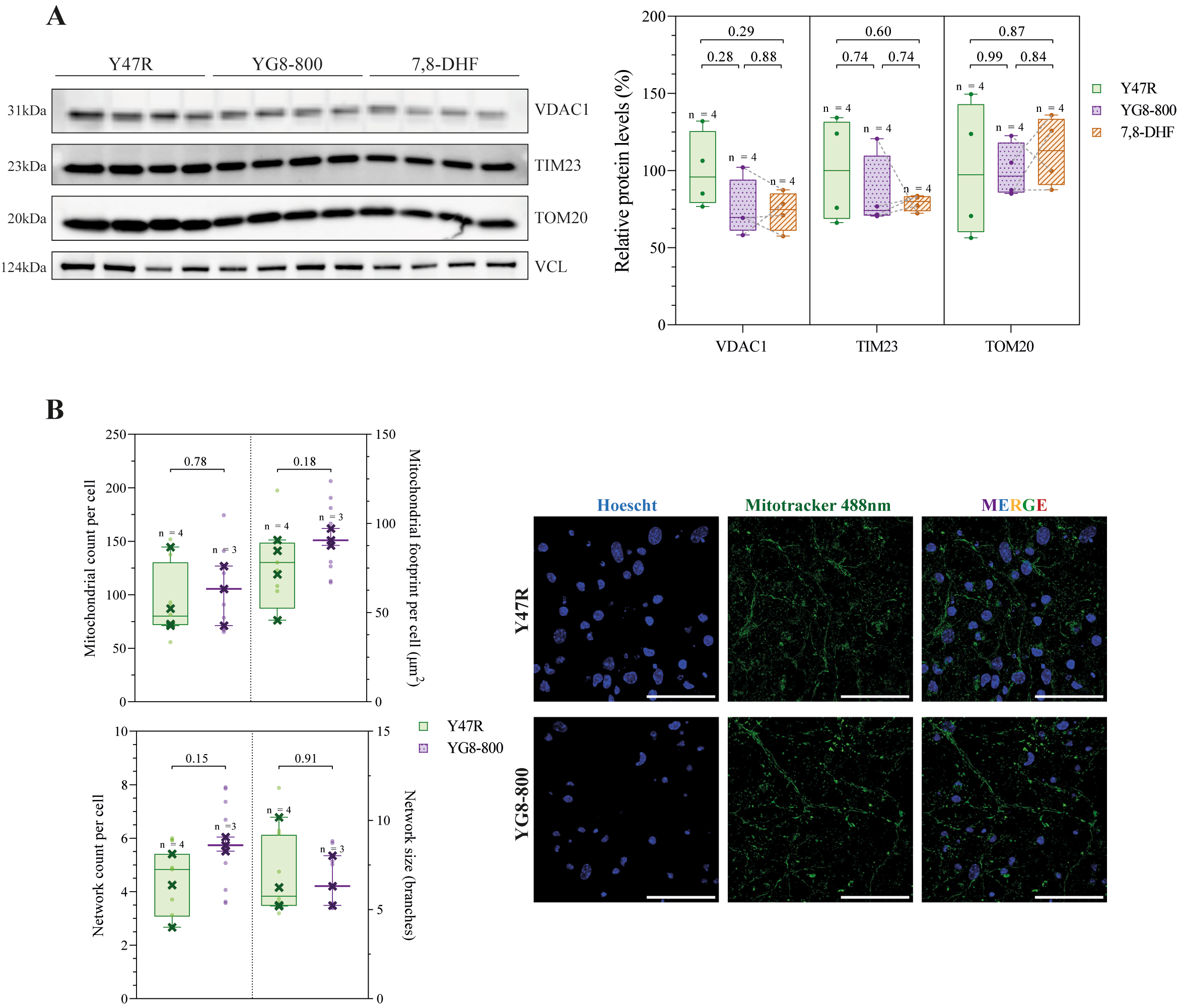

Supplement: Supplementary file 5 — Measurement of mitochondrial mass. A—Relative protein levels of the mitochondrial proteins VDAC1, TIM23 and TOM20, quantified by Western Blot, from Y47R and YG8-800 untreated or 7,8-DHF-reated CGNs. Dashed lines connect matched untreated and 7–8-DHF-treated YG8-800 samples. Representative immunoblots are shown on the left. B – Analysis of the mitochondrial network, using the ImageJ plugin Mitochondrial Network Analysis (MiNA), upon green mitotracker staining. Representative live-cell images are shown on the right. Dots represent the quantification from a single field, and crosses represent the average for each mouse, Data were analysed using a paired Student’s T-Test between untreated and 7,8-DHF-treated YG8-800 CGNs (within-mouse comparison) and unpaired Student’s T-Test between Y47R and untreated and 7,8-DHF-treated YG8-800 CGNs. N number and p values are shown in each graph. VCL (vinculin) was used as a loading control in A. In blue, the nuclear marker, Hoestch, in B. (PNG 1.15 MB) [file 12035_2026_5856_Fig7_ESM.png]

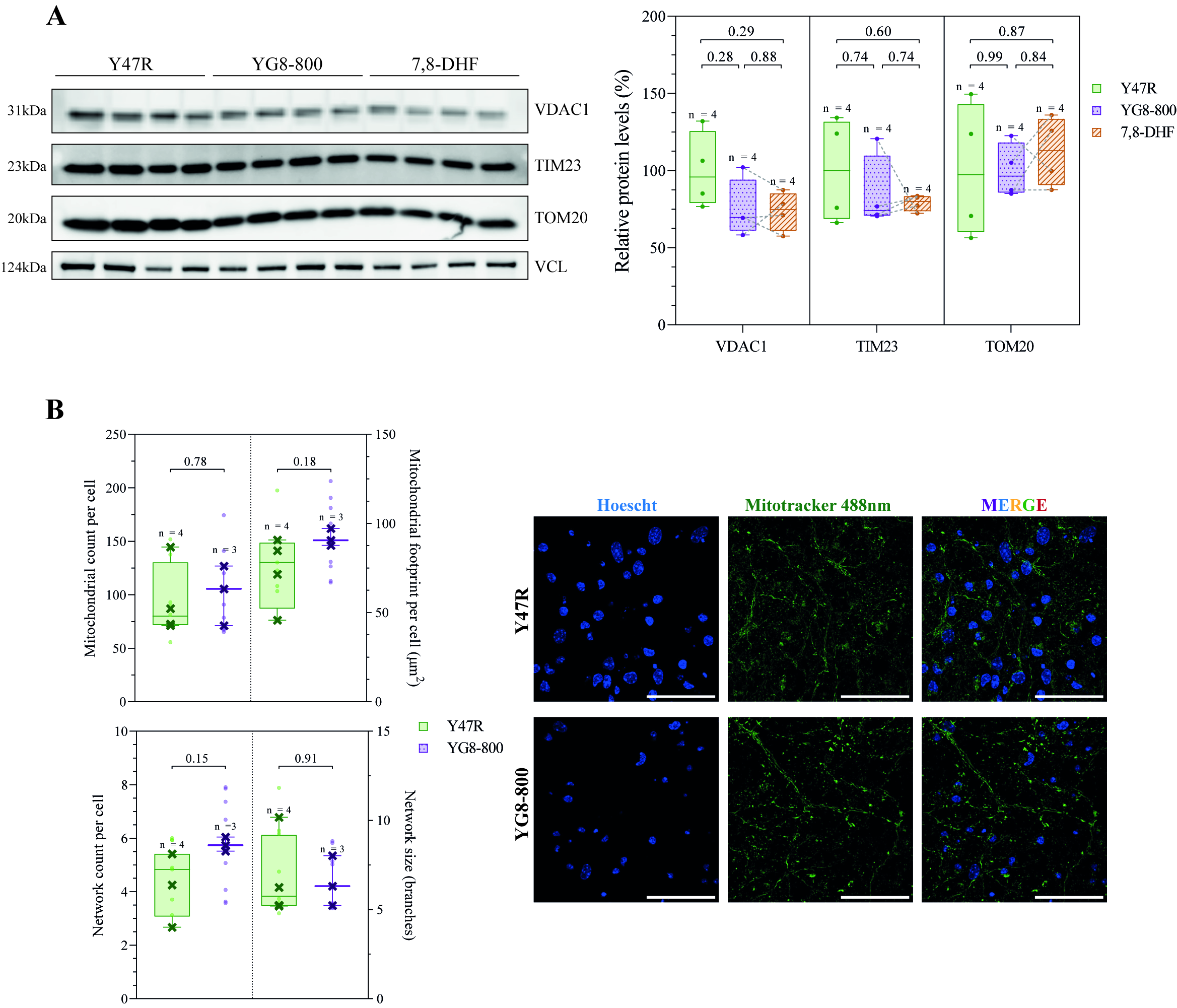

Supplement: Supplementary file 6 — High Resolution Image (TIF 21.8 MB) [file 12035_2026_5856_MOESM4_ESM.tif]
