# Supplementary material for: The TRKB Agonist 7,8-dihydroxyflavone Alleviates DNA Damage and Apoptosis in a Neuronal Cell Model of Friedreich’s Ataxia
Source: Mol Neurobiol. 2026 Apr 22;63(1):580. doi: 10.1007/s12035-026-05856-2 (PMC13102869; doi:10.1007/s12035-026-05856-2)
Supplement: Supplementary file 7 — (DOCX 14.0 KB) [file 12035_2026_5856_MOESM5_ESM.docx]

**Supplementary file S5: Supplementary methods**

**Mitotracker staining and mitochondrial network analysis**

The commercial 488 green mitotracker dye (ThermoFisher Scientific M7514) was used to stain mitochondrial network of live cells. Cells were cultured in µ-Slide 8 well plates (IBIDI, Cat. No. 80806) and incubated 30 min with 1 μM of green mitotracker dye at 37ºC. Then, they were washed once with PBS, incubated 15 min with Hoechst (1:1000) at 37ºC and washed again with PBS. Finally, randomly selected fields were captured using a Laser Scanning Confocal Microscope LSM800, with cells maintained at 37ºC and 5% CO_2_. The mitochondrial network analysis was done using the ImageJ plugin Mitochondrial Network Analysis (MiNA).

**Western blotting**

Please refer to main text for description.

Antibodies used in supplementary files:

| Protein | Source | Reference | Dilution |
| --- | --- | --- | --- |
| TIM23 | Mouse | BD Biosciences #611222 | 1:1000 (WB) |
| TOM20 | Rabbit | Santa Cruz biotechnology #sc11415 | 1:1000 (WB) |
| TRKB | Rabbit | Santa Cruz biotechnology #sc12 | 1:1000 (WB) |
| pTRKB-Y705 | Rabbit | GenScript A01186 | 1:1000 (WB) |
| VCL | Mouse | Sigma-Aldrich #CP74 | 1:2000 (WB) |
| VDAC1 | Mouse | Abcam #ab14734 | 1:1000 (WB) |

**Metabolic activity and cell viability assays**

Please refer to main text for description.
